# Syphilitic Sarcocele

**Published:** 1866-07

**Authors:** 


SYPHILITIC SARCOCELE. SLOUGHING OF THE SCROTUM. REMOVAL OF THE GLAND. NITROUS-OXIDE. ——, æt. 22 The patient contracted chancre three years ago, which was followed by inguinal enlargement and profuse skin eruption. Ulceration of his nose is a prominent symptom, and the part has assumed the flattened appearance consequent upon the loss of its bony structure. About a year since, the right testicle began to enlarge, became heavy, very hard, and, at times, painful. Six weeks ago, the testicle increased enormously in size, the skin covering it sloughed, leaving the organ entirely denuded. There is a fetid discharge, which weakens him very much. In this disease we find the organ infiltrated with a yellowish lymph, which is deposited in and around the tubes, finally obliterating the normal structure. The parts here have sloughed to such an extent as to leave no chance for the organ being covered by skin. In consequence of this, the great discharge, and the disorganization of the part, we shall remove it. The nitrous-oxide was inhaled, perfect anæsthesia being induced, an incision was made running up the cord, which was turned out and divided. The hæmorrhage being controlled with one acupressure needle. —*Medical & Surgical Reporter.*

